# Acclimation of *Chlamydomonas reinhardtii* to extremely strong light

**DOI:** 10.1007/s11120-020-00802-2

**Published:** 2020-12-06

**Authors:** Olli Virtanen, Sergey Khorobrykh, Esa Tyystjärvi

**Affiliations:** Department of Biochemistry/Molecular Plant Biology, University of Turku, 20014 Turku, Finland

**Keywords:** *Chlamydomonas reinhardtii*, Photosystem II, PSII, High light, Extreme light, Acclimation, Light stress

## Abstract

Most photosynthetic organisms are sensitive to very high light, although acclimation mechanisms enable them to deal with exposure to strong light up to a point. Here we show that cultures of wild-type *Chlamydomonas reinhardtii* strain *cc124*, when exposed to photosynthetic photon flux density 3000 μmol m^−2^ s^−1^ for a couple of days, are able to suddenly attain the ability to grow and thrive. We compared the phenotypes of control cells and cells acclimated to this extreme light (EL). The results suggest that genetic or epigenetic variation, developing during maintenance of the population in moderate light, contributes to the acclimation capability. EL acclimation was associated with a high carotenoid-to-chlorophyll ratio and slowed down PSII charge recombination reactions, probably by affecting the pre-exponential Arrhenius factor of the rate constant. In agreement with these findings, EL acclimated cells showed only one tenth of the ^1^O_2_ level of control cells. In spite of low ^1^O_2_ levels, the rate of the damaging reaction of PSII photoinhibition was similar in EL acclimated and control cells. Furthermore, EL acclimation was associated with slow PSII electron transfer to artificial quinone acceptors. The data show that ability to grow and thrive in extremely strong light is not restricted to photoinhibition-resistant organisms such as *Chlorella ohadii* or to high-light tolerant mutants, but a wild-type strain of a common model microalga has this ability as well.

**Electronic supplementary material:**

The online version of this article (10.1007/s11120-020-00802-2) contains supplementary material, which is available to authorized users.

## Introduction

Light is the driving force of photosynthesis but also a stress factor affecting both photosystems. Photosystem II (PSII) is particularly susceptible to light-induced damage, and the rate of damage is proportional to light intensity (Tyystjärvi and Aro 1996). Photoinhibition is counteracted by concurrent repair, and several biochemical mechanisms offer partial protection (for review, see Tyystjärvi 2013), but in spite of the protective mechanisms, light intensities far above saturation are expected to lower the number of active PSII units and thereby cause decrease in the photosynthetic rate. Furthermore, reactive oxygen species produced in very high light are also expected to cause oxidative damage, and oxidative repression of translational elongation (Nishiyama Y et al. 2004) directly interfering with the repair of photoinhibitory damage.

Green microalgae that live in surface waters are exposed to periods of high light. Earlier experiments have shown that *C. reinhardtii*, when exposed to strong light for some time, can form cultures that can be continuously grown in strong light (Förster et al. 2005). Mutation leading to a high-light tolerant phenotype is an obvious possible mechanism for these changes, and high-light tolerant mutants of *C. reinhardtii* have been isolated (Förster et al. 2005; Schierenbeck et al. 2015), and at least the high-light tolerant *hit2*-mutant has properties that enables it to tolerate photoinhibition of PSII (Virtanen et al. 2019). We exposed the wild-type strain *cc124* of *C. reinhardtii* to extremely strong light (EL) and found that the alga regularly switches to a phenotype tolerant to EL after only a few days of exposure to EL conditions. The change is too rapid and frequent to be caused by random mutations, which prompted us to explore acclimatory changes in photoprotective mechanisms.

Studies of both damage and acclimation to high light have largely focused on PSII because PSII is much more sensitive to high light than PSI in strong continuous light (Tyystjärvi et al. 1989; Sonoike 2011), PSII is the major producer of the harmful singlet oxygen (^1^O_2_) in photosynthetic organisms (Hideg and Vass 1995; Fufezan et al. 2002; Krieger-Liszkay 2005; Krieger-Liszkay et al. 2008; Cazzaniga et al. 2012; Telfer 2014, for recent review on singlet oxygen see Dimitrieva et al. 2020), and because the rapid turnover of the D1 protein in high light makes PSII specifically sensitive to damage to the translation machinery (Nishiyama Y et al. 2001). The short lifetimes of excited chlorophylls in PSI (for review, see Chukhutsina et al. 2019) do not favor production of ^1^O_2_ in PSI antenna. Instead of producing ^1^O_2_, PSI can reduce oxygen to superoxide in Mehler's reaction (Lima-Melo et al. 2019). Reactive oxygen species are linked to inactivation of PSI (for review, see Sonoike 2011; for their importance in fluctuating light see Sejima et al. 2014) but in high light, the primary donor of PSI tends to stay oxidized, which protects PSI against damage (for review, see Shimakawa and Miyake 2018). Furthermore, as Mehler's reaction and inactivation of PSI require PSII electron transfer, inactivation of PSI is not directly dependent on light. Moreover, PSI is also protected by several different mechanisms regulating electron transfer (for review, see Tikkanen and Aro 2014) and by downregulation of PSII in high light (Ivanov et al. 1998; Lima-Melo et al. 2019). For these reasons, our focus will be on PSII, although we cannot rule out additional acclimation responses that might specifically protect other parts of the photosynthetic machinery, especially PSI, during exposure to EL.

Non-photochemical quenching of absorbed excitation energy (NPQ) is a major PSII-specific mechanism that helps to avoid the damage caused by high light (Wobbe et al 2016). However, NPQ mechanisms protect the system only up to a degree (Sarvikas et al. 2006; Havurinne et al. 2019). In contrast to plants, where the most rapidly induced component of NPQ is ∆pH dependent heat dissipation (qE), the most rapid response to high light in green algae is a state transition leading to qT-type NPQ. Light-Harvesting Complex Stress-Related proteins 1 and 3 (LHCSR) are constitutively present in *C. reinhardtii* cells grown in the light (Nawrocki et al. 2019), and they are activated by light-induced decrease in lumenal pH (Bonente et al. 2011; Liguori et al. 2013; Kondo et al. 2017; Tian et al. 2019). Active LHCSR3 induces rapid decoupling of LHCII from PSII shortly after the beginning of high-light exposure (Roach and Na 2017). Chlorophylls (Chls) of decoupled LHCII have a very short excitation lifetime and function as excitation energy sinks (Ünlü et al. 2014). The decoupling also efficiently decreases the functional antenna size of PSII (Tian et al. 2019). These mechanisms are considered to protect the system efficiently although the LHCSRs are also required for the formation of qE within several hours of high-light exposure (Peers et al. 2009; Allorent et al. 2013). Furthermore, acidification of the thylakoid lumen activates the STT7 kinase that phosphorylates LHCII that then decouples from PSII and moves to serve PSI like in higher plants. In *C. reinhardtii*, 80% of LHCII can disassociate from PSII (Delosme et al. 1996) while only 15% is estimated to move between photosystems in *A. thaliana* (Allen 1992). However, in *C. reinhardtii* only a small part of LHCII that is decoupled from PSII transfers excitation energy to PSI (Nagy et al. 2014; Ünlü et al. 2014), further emphasizing the photoprotective role of qT in *C. reinhardtii*. In addition to the LHCSR-dependent excitation energy quenching, *C. reinhardtii* downregulates the amount of Chl per cell and upregulates the carotenoid-to-Chl ratio upon long exposure to high light (Virtanen et al. 2019). These mechanisms decrease the incoming excitation of PSII and promote quenching of reactive oxygen species by carotenoids. In addition, the PSI to PSII ratio is downregulated during acclimation to high light in *C. reinhardtii*; the advantage of this response, however, is not known (Bonente et al. 2012; Virtanen et al. 2019).

Generally, high-light-tolerating or slow-photoinhibition phenotypes produced by mutations are relatively mild. For example, at the photosynthetic photon flux density (PPFD) of 1250 μmol m^−2^ s^−1^, the continuous productivity of the high-light tolerant *hit2* mutant of *C. reinhardtii* (Schierenbeck et al. 2015) with a mutation in the *Cr-COP1* gene involved in ultraviolet signaling (Tilbrook et al. 2016), is only one fourth higher than that of the wild type (Virtanen et al. 2019). In addition, the redox potential of the *Q*_A_/*Q*_A_^−^ pair determines the probability of formation of a triplet state of the primary donor by PSII recombination reactions, thereby affecting the probability of formation of the poisonous singlet oxygen (^1^O_2_) (Krieger-Liszkay et al. 2008). In the cyanobacterium *Synechococcus elongatus*, the A249S mutation of the D1 protein makes the redox potential of the *Q*_A_/*Q*_A_^−^ pair more positive and causes a lowering of approximately one fourth in the rate of photoinhibition compared to the wild type (Fufezan et al. 2007). Because the rate constant of photoinhibition is directly proportional to light intensity (Tyystjärvi and Aro 1996), protection by one fourth suggests that the mutants would tolerate approximately one fourth higher light intensity than the wild type.

In the present study, we show that wild-type cells of *C. reinhardtii* regularly develop a capability to grow rapidly at PPFD 3000 µmol m^−2^ s^−1^ in mineral medium. This PPFD is approximately 1.5 times full sunlight and 10–20 times as high as usually applied in laboratory cultivation of *C. reinhardtii*. Photosynthetic properties and ^1^O_2_ production of cells growing in this extremely strong light were compared to those growing at moderate PPFD to pinpoint the features that might cause the observed tolerance.

## Materials and methods

### Algal strain and growth conditions

All experiments were conducted with the *cc124* wild-type strain of *C. reinhardtii*. The cells were maintained on Tris–Acetate–Phosphate (TAP) plates (Gorman and Levine 1965) and transferred to a liquid, photoautotrophic high salt (HS) medium (Sueoka 1960) prior to the experiments. In this liquid HS medium, the cells were first kept in pre-culture conditions in moderate light conditions (27 °C, PPFD 100 µmol m^−2^ s^−1^) to acclimate the cells to photoautotrophy. The cells were grown in 1% CO_2_ to enhance photosynthetic growth during this precultivation.

### Extreme-light growth experiment

Experiments testing the ability of the cells to grow in extremely strong light were done by cultivating two types of cultures in these conditions. Two types of cultures prepared for growing in extreme light conditions were isolated subpopulations that were populations inoculated from the original culture, and populations originating from single, individual cells of the original culture obtained via dilution and plating on solid HS medium. These isolated subpopulations or single-cell-originating cultures of *cc124,* as indicated, were then first cultivated in liquid HS medium in pre-culture conditions (27 °C, PPFD 100 µmol m^−2^ s^−1^, 1% CO_2_) and then diluted to OD_730_ (optical density at 730 nm) of 0.01. The diluted 45 ml cultures in HS medium were transferred to PPFD 3000 µmol m^−2^ s^−1^, 26 °C and ambient air (hereafter referred to as extreme light, EL) in 100 ml Erlenmeyer flasks. Mixing was provided with an orbital shaker. Combination of white 30 W LEDs (LED Energie, model no. 6208 5667)) and 10 W LEDs (IKEA, Product no. LED1506R10) were used to create the extremely strong light (see Fig. S1 for the illumination spectrum in the EL conditions). Growth was monitored for 96 h by daily measurements of OD_730_, and cultures whose OD_730_ had increased to 0.05 were used for further experiments. The EL experiment was conducted with 30 biological replicates of both isolated subpopulation and single cell types of inocula.

### Pigment concentrations

Samples for pigment extraction were taken from cultures diluted to OD_730_ of 0.5. 1 ml aliquots were centrifuged for 10 min at 14,000×*g* and resuspended in 1 ml of methanol. After thorough mixing, the pigments were extracted in cold (+ 4 °C) and darkness. After 24 h of extraction, the samples were centrifuged for 10 min at 14,000×*g*, after which absorbance of the supernatant was measured at 470, 652.4 and 665.2 nm, and the pigments were quantified according to Wellburn (1994).

### Low temperature fluorescence spectra

The samples for fluorescence emission were taken directly from EL and control cultures and stored at − 80 °C until measured. The samples were diluted to the Chl concentration of 1.5 µg Chl ml^−1^ and a final volume of 50 µl just prior to the measuring the spectra in vivo. Frozen samples were illuminated at liquid nitrogen temperature (− 196 °C) with 442 nm blue light, and fluorescence emission was measured with a QEPro spectrometer (Ocean Insight, Ostfildern, Germany).

### Plastoquinone measurement

The total amount of plastoquinone (PQ) was determined from EL and control cells with high performance liquid chromatography (Khorobrykh et al. 2020) by utilizing the detection of fluorescence of reduced plastohydroquinone (PQH_2_) at 330 nm with excitation wavelength at 290 nm. The preparation of the calibration standard has been published earlier (Khorobrykh et al. 2020). Quantities obtained were then normalized to the Chl concentrations, measured separately from all three biological replicates.

### Quantification of PSII and PSI

Proteins were extracted from approximately 20 million cells, collected via centrifugation (12,000×*g*, 10 min) and resuspended in protein extraction buffer (50 mM Tris–HCl; pH 8, 2% SDS, 10 mM EDTA). After resuspension, the cells were frozen in liquid nitrogen, followed by thawing in a 45 °C water bath. This was repeated three times in rapid succession, after which the debris was removed via centrifugation (15,000×g, 5 min).

The relative amounts of PSII and PSI were estimated from Western blots with antibodies for two of their core proteins, CP43 and PsaA, respectively. Proteins were first separated with SDS-PAGE, using 1 µg (for CP43) or 2 µg (for PsaA) of protein per well. These amounts of protein were found to be optimal for detection through dilution series. Primary antibodies for CP43 (Agrisera, product no. AS06 110) and PsaA (Agrisera, product no. AS06 172) were used in concentrations of 1:6000 and 1:5000, respectively. The secondary antibody, goat-anti rabbit IgG (H + L), alkaline phosphatase conjugate (Life technologies, REF G21079) was used in final concentration of 1:50 000 and the binding was detected via luminescence caused by alkaline phosphatase. Relative amounts of the proteins were calculated from signal intensities, quantified with the image processing software Fiji (Fiji Is Just ImageJ, v. 1.52).

### Oxygen evolution measurements

The light-saturated rate of oxygen evolution (under PPFD 2000 µmol m^−2^ s^−1^) was measured from 1 ml samples of intact cells with a Clark-type oxygen electrode (Hansatech Instruments Ltd, Norfolk, United Kingdom) at 25 °C in HS medium. For comparison of electron acceptors, the samples were diluted to OD_730_ of 0.5. Artificial electron acceptor 2,6-dimethylbenzoquinone (DMBQ, 0.5 mM); 2,5-dichloro-1,4-benzoquinone (DCBQ, 0.5 mM); ferricyanide (FeCy, 0.5 mM)), or an inhibitor of electron transfer (2,5-dibromo-6-isopropyl-3-methyl-1,4-benzoquinone (DBMIB, 0.5 µM), as indicated, was added just before the measurement.

Thylakoids were isolated as described previously in Virtanen et al. (2019). The chlorophyll concentration of the thylakoid isolates was determined spectrophotometrically according to Porra et al. (1989). Isolated thylakoids were stored at − 70 °C till measurements. Oxygen evolution of the isolated thylakoids was measured as in vivo except that thylakoids were diluted in PSII measuring buffer (40 mM HEPES–KOH pH 7.6; 0.33 M sorbitol; 5 mM MgCl_2_; 5 mM NaCl; 1 M glycine betaine; 1 mM KH_2_PO_4_; 5 mM NH_2_Cl) in final volume of 1 ml and chlorophyll concentration of 5 µg ml^−1^. DMBQ, DCBQ and FeCy were applied in the same concentrations as used for the in vivo-measurements.

### Fluorescence measurements

Chl *a* fluorescence decay after a single turnover flash (60% of maximum voltage corresponding to PPFD 10^5^ µmol m^−2^ s^−1^, as reported by manufacturer, flash duration 30 µs) was measured with a Superhead high-sensitivity detector, connected to an FL200/PS control unit (Photon Systems Instruments, Drásov, Czech Republic) from 2 ml samples with 5 µg ml^−1^ of Chl at 25 °C in the presence and absence of 10 µM 3-(3,4-dichlorophenyl)-1,1-dimethylurea (DCMU), as indicated. Before measurement, the samples were dark-incubated for 15 min in ambient air. Each measurement lasted for 120 s, the first data point was recorded 300 µs after the single turnover flash, and three independent biological replicates were measured for all conditions. Results in the presence of DCMU were fitted to a first-order reaction to obtain the rate constant of recombination reactions. *Copasi*-software (Hoops et al. 2006) was used for fitting.

Fluorescence induction was measured both in the absence and in the presence of 10 µM DCMU from intact *C. reinhardtii* cells with AquaPen fluorometer (AquaPen AP100, Photon Systems Instruments, Drásov, Czech Republic). 2 ml samples with Chl concentration of 1 µg ml^−1^ were dark-incubated for 15 min in ambient air, and the intensity of the actinic light was set to 40% of the maximum of the instrument. Actinic light intensity and Chl concentration were optimized in preliminary experiments to obtain a valid signal. DCMU, when used, was added before the 15 min dark-incubation in the final concentration of 10 µM. Three independent biological replicates were measured from all conditions.

### Thermoluminescence

Thermoluminescence was measured in vivo with an apparatus described before (Tyystjärvi et al. 2009) from 200 µl samples containing 3.1 µg Chl. For Q band measurements, 10 µM DCMU was added. The samples were dark-incubated at 20 °C for 5 min and then cooled to either − 10 °C for B band measurement or -20 °C for Q band measurement, as indicated. The frozen sample was charged with a single turnover flash (*E* = 1 J) from a Xenon flash lamp and photon emission was recorded during warming to 60 °C with a heating rate of 0.66 °C s^−1^. Simulation of thermoluminescence was done with the *Copasi* software. In the simulation, thermoluminescence intensity during heating from 274 to 340 K with the rate of 0.66 K s^−1^ was simulated as the rate of a first-order reaction whose rate constant is *s* × exp(− 501 meV/(*k*_b_ × (274 K + 0.66 K s^−1^ × *t*)), where *s* is a pre-exponential factor, *k*_b_ is Boltzmann's constant, *β* is the heating rate and *t* is time from start of heating. 501 meV is the activation energy.

### Singlet oxygen production

^1^O_2_ production by *C. reinhardtii* was measured in vivo using Singlet Oxygen Sensor Green (SOSG) (Invitrogen by ThermoFischer Scientific). Cell cultures were concentrated through gentle centrifugation (100×*g*, 1 min) to a Chl concentration of 50 µg ml^−1^. SOSG was added to 350 µl samples in final concentration of 50 µM. ^1^O_2_ production was induced by illuminating the samples with red light, PPFD 2000 µmol m^−2^ s^−1^, obtained from a slide projector and a 650 nm long-pass filter (Corion LL650, Newport Corp.). SOSG fluorescence was recorded every 10 min by switching off the 650 nm illumination and exciting with light from a slide projector filtered through a 500 nm narrow band filter (Ealing Electro-Optics, Inc. Holliston, MA, USA) and a 600 nm short-pass filter (Corion SL600, Newport Corp.). Fluorescence emission was recorded with a QEPro spectrometer. The average rate of increase in SOSG fluorescence between 535 and 540 nm during three consecutive 10 min red light illumination periods was taken as a relative rate of ^1^O_2_ production. Values were then averaged between three independent biological replicates. Control measurements were done from illuminated samples containing no algal cells and from algal cell samples incubated in the dark.

### Photoinhibition of PSII

Light-induced loss of PSII activity was measured from algal samples diluted to OD_730_ of 0.5. A 10 ml sample was subjected to strong light (PPFD 950 µmol m^−2^ s^−1^) from a 1000 W low-pressure Xenon lamp (201-1 k, 1000 W, Science tech inc, London, ON, Canada) equipped with an ultraviolet protection film (Long Life for Art, Eichstetten, Germany) and a 9-cm water filter to remove heat. The light path in the sample was 7 mm and the temperature was maintained at 25 °C throughout the experiment. The light-saturated rate of oxygen evolution (H_2_O to DMBQ) was measured from a 1 ml aliquot before illumination and during illumination every 10 min. Lincomycin, when present, was used at 0.5 mg ml^−1^ and added before the measurement of the control rate of oxygen evolution. For comparison of the rates, the measured oxygen evolution rates were first divided by the Chl concentration and then by the control value of the respective sample. The loss of oxygen evolution in the presence of lincomycin was fitted to a first-order reaction equation to obtain the rate constant of the damaging reaction of photoinhibition of PSII (k_PI_). The measurements were conducted with three independent biological replicates from all conditions.

## Results

### Variation within the cultures contributes to survival and growth in extreme light

**Fig. 1:**
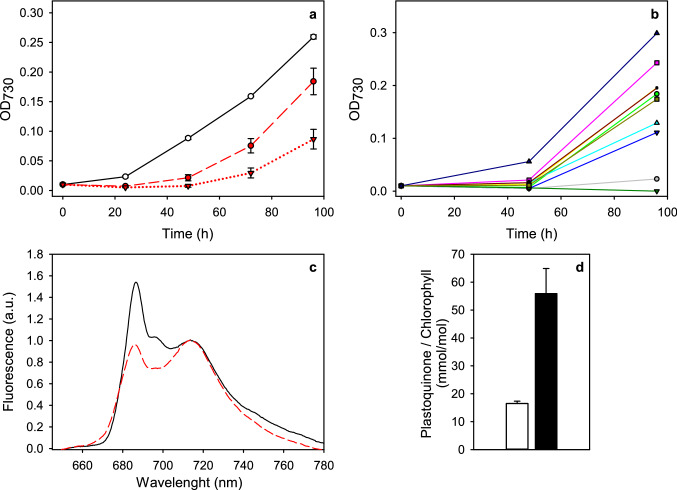
**a** Growth of cultures of *C. reinhardtii* strain *cc124* in control conditions (open circles, solid line) and two types of cultures in EL: isolated subpopulations (red circles, red dashed line) and single-cell-originating inocula (red triangles, red dotted line). **b** the growth of individual, EL-categorized, single-cell-originating cultures, after being cultured in moderate PPFD of 100 µmol m^−2^ s^−1^ for 1 week after initial EL acclimation. The growth of the cultures was determined spectrophotometrically as increase in optical density at 730 nm. The inoculum density was 0.01, after which the cultures were kept for 96 h in the light conditions described. The cells were grown in 45 ml of photoautotrophic medium at room temperature and ambient atmosphere on a shaker. The curves in **a** are averaged from 3 (control) or 30 (EL cultures) independent biological replicates and the error bars show SEM. The curves in **b** represent observations from individual cultures. **c** Fluorescence emission spectrum of control (solid, black line) and EL cells (dashed, red line) measured at 77 K. The samples taken directly from growth conditions were stored at − 80 °C and diluted to 1.5 µg Chl ml^−1^ and final volume 50 µl upon measurement. Fluorescence was measured with QEPro spectrometer with 442 nm excitation. The data were normalized to the value at 713 nm. **d** Total amount of PQ in control (white bar) and EL (black bar) cells, normalized to Chl concentration. The total amount of PQ was measured with a HPLC method (Khorobrykh et al. 2020) from cultures that had reached the end of exponential growth phase; the Chl concentrations for the normalization were measured spectrophotometrically from methanol extracts of the cultures. All the data in **c** and **d** are averaged from three independent biological replicates and the error bars show SD

Transfer of *C. reinhardtii* cultures from PPFD 100 to 3000 μmol m^−2^ s^−1^ led first to death of some cells, as indicated by a decrease in OD_730_ during the first 24 h in EL (Fig. 1a). Thereafter, however, most cultures resumed growth. Isolated subpopulations were more likely to acclimate to extreme light within 96 h than cultures originating from single cells (Table 1). After 96 h in EL, the average OD_730_ of single-cell-originating cultures was 0.09 ± 0.02 (Fig. 1a), whereas the average OD_730_ of isolated subpopulations was 0.18 ± 0.02. These numbers only include cultures that reached the OD_730_ level of 0.05 within 96 h of transfer to EL, and only such cultures were used in further experiments. However, we noted that practically all EL-exposed cultures eventually started to grow if the exposure to EL continued. After 96 h, control cultures that were kept in moderate light had reached the OD_730_ of 0.259 ± 0.003, a significantly higher cell density than either of the EL grown cultures (*P* ≪ 0.05).

**Table 1:** Statistics of *C. reinhardtii *cultures grown in EL (PPFD 3000 µmol m^−2^ s^−1^). The EL cultures were started as isolated subpopulation of the maintenance culture or from inocula grown separately starting from single cells, as indicated. Before shifting to EL, all cultures were diluted to OD_730_ of 0.01

	% of cultures that reached OD_730_ of 0.05 in 24 h	% of cultures that reached OD_730_ of 0.05 in 48 h	% of cultures that grew over OD_730_ of 0.05 in 96 h
Cultures originating from single cells	0	3.3	56.7
Isolated subpopulations	0	13.3	80
HL-categorized cultures reintroduced to extreme light after 1 week in moderate light	–	10	80

We also tested if the acclimation response was permanent by transferring 10 EL cultures to low light for 7 days and then re-introducing them to the EL conditions. Not all cultures grew in the same, EL-tolerating manner as previously (Fig. 1b). On average, the cultures that withstood the re-introduction to extreme light had the OD_730_ of 0.172 ± 0.09, similar as the density the isolated subpopulations at the same time point after onset of the first EL treatment.

### Acclimation lowers the amount of Chl but does not affect the amount of carotenoids

**Fig. 2:**
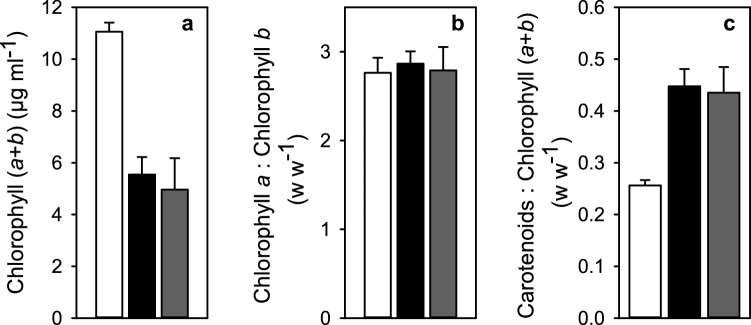
**a** Total concentration of Chls *a* and *b*, **b** ratio of Chls *a* and *b* and **c** ratio of total carotenoids to Chls, measured from pre-condition grown control (white bars), and EL grown isolated subpopulation (black bars) and single-cell-originating cultures (grey bars) of *C. reinhardtii*. The pigments were extracted in cold (+ 4 °C) and darkness via methanol extraction from cultures with OD_730_ of 0.5. The samples were taken directly from the cultures grown in control or EL conditions. The bars are averaged from three (control) or 15–17 (EL cultures) biological replicates and the error bars show SD

The sum of Chls *a* and *b* in cell cultures of the same OD_730_ decreased to one half during the acclimation to EL (Fig. 2a), indicating a decrease in Chl per cell. The Chl *a*/*b* ratio did not change in response to the acclimation (Fig. 2b). In same cultures, the amount of carotenoids per cell remained rather constant during acclimation to EL, which led to doubling of the carotenoid-to-Chl ratio (Fig. 2c). The pigment analysis showed no differences between EL-exposed cultures originating from the two types of inocula (Fig. 2).

As all EL cultures, whether they originated from single cells or isolated subpopulations, obviously shared the same phenotype, EL cultures for all further experiments were prepared with the isolated subpopulation method.

### Ratio of PSII to PSI fluorescence emission decreases during EL acclimation

Fluorescence emission was measured at the temperature of 77 K to see how the stoichiometry of PSII and PSI behaves when the cells acclimate to EL. The results (Fig. 1c) show that the ratio of fluorescence originating from PSII to fluorescence from PSI decreased from 1.5 in control cells to 0.95 in EL cells as result of the acclimation.

### PQ-to-Chl ratio is higher in EL than in control cells

PQ is an electron carrier molecule in the thylakoid membrane but in plants (Kruk and Karpinski 2006) and cyanobacteria (Khorobrykh et al. 2020), a large part of PQ is located outside of the thylakoid membrane and does not take part in electron transfer. In plants, this non-photoactive PQ is found in plastoglobuli and in the inner chloroplast envelope. We measured the amount of PQ and found that the PQ-to-Chl ratio was approximately threefold as high in EL as in control cells (Fig. 1d). Comparison of Figs. 1d and 2a reveals that the amount of PQ per cell is higher in EL than in control cells, as the ratio of the chlorophyll contents of control and EL cells is approximately two.

### Number of photosystems decreases in response to the EL acclimation

**Fig. 3:**
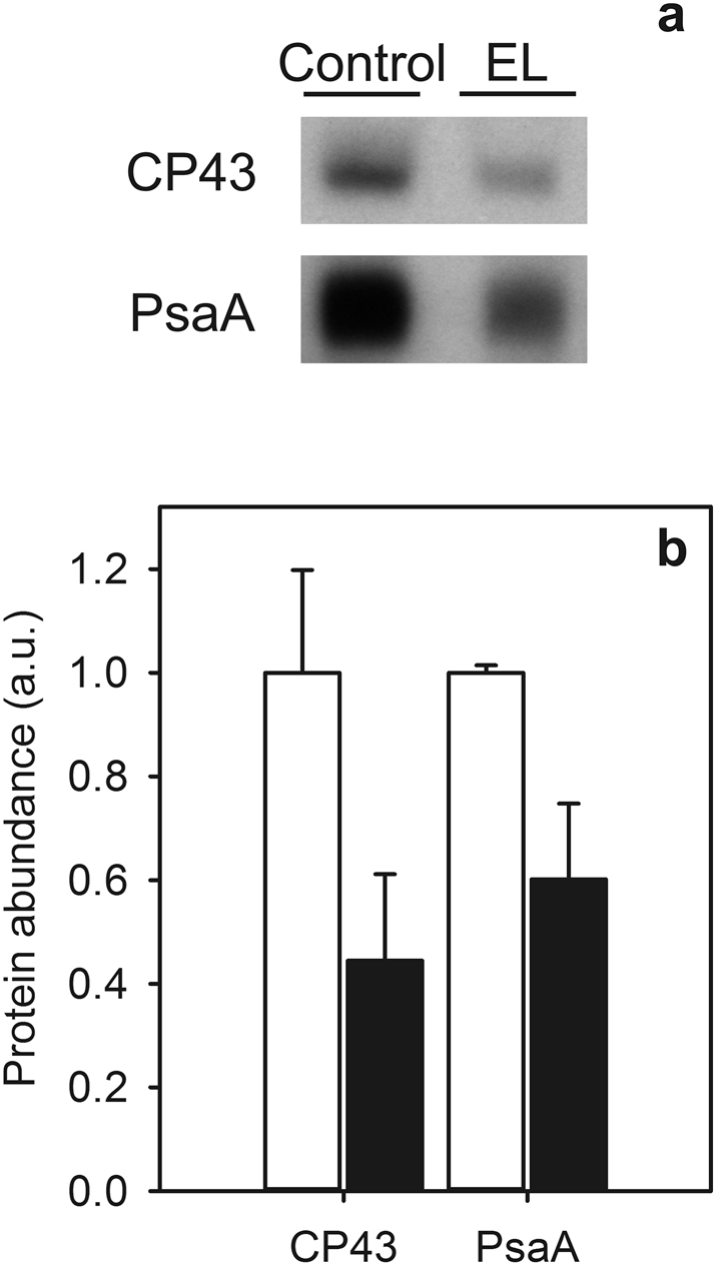
Detection of CP43 and PsaA proteins on a film (**a**) and quantification of these proteins (**b**) from control (white bars) and EL (black bars) cells. Western blotting was done from extracted total soluble proteins, and 1 µg (CP43) or 2 µg (PsaA) of total proteins were loaded to SDS-PAGE per well. Binding of the primary and secondary antibodies was visualized via luminescence emitted by alkaline phosphatase. The signals were normalized to the average of signals originating from control samples of the respective western blot. Each bar represents an average of three biological replicates and the error bars show SD

Decrease in total number of photosystems is a known response to increasing light intensity, and PSI has been shown to be more heavily downregulated than PSII during high-light cultivation of *C. reinhardtii* (Bonente et al. 2012). In the EL acclimation, the number of both photosystems was found to decrease. Judging from the quantification of Western blots of the CP43 and PsaA proteins, the PSII and PSI contents of the EL cells were 44.5 ± 16.7% and 60.2 ± 14.6% of the control cells, respectively (Fig. 3). These numbers match with the overall reduction in the amount of chlorophyll to about one half during EL acclimation (Fig. 2a).

### EL acclimated cells do not reduce artificial quinone electron acceptors efficiently

PSII can reduce a range of electron acceptors in addition to the natural acceptor PQ, and we hypothesized that acclimation to EL might change the affinity of PSII to artificial electron acceptors. To test this, the light-saturated rate of oxygen evolution was measured from control and EL cells using different electron acceptors. To avoid changes in the cultures after the EL treatment, the measurements were done immediately after removing an aliquot from the EL culture and diluting to the standard OD_730_ of 0.5. The time-consuming quantification of Chl was done subsequently from parallel aliquots, and the average Chl concentrations of the control and EL samples were 11.08 ± 0.40 and 3.89 ± 0.33 µg ml^−1^, respectively.

**Fig. 4:**
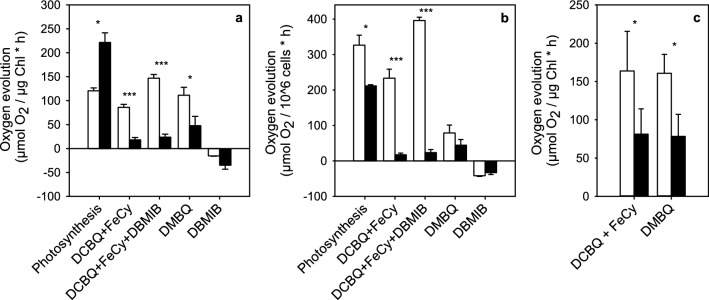
PSII activity, measured as light-saturated oxygen evolution from control (white bars) and EL samples of *C. reinhardtii* (black bars) both in vivo (**a**, **b**) and in isolated thylakoids (**c**) with different electron acceptors and one inhibitor of electron transfer, normalized to Chl (**a, c**) or cell (**b**) concentrations. Light-saturated oxygen evolution was measured at PPFD 2000 µmol m^−2^ s^−1^ from cultures with OD_730_ of 0.5. The cultures were grown in PPFD of either 100 or 3000 µmol m^−2^ s^−1^ and 1 ml samples were used in measurement. Isolated thylakoids were used in final chlorophyll concentration of 5 µg ml^−1^. The concentrations of artificial electron acceptors (DCBQ, FeCy and DMBQ) were 0.5 mM and the inhibitor (DBMIB) was added at the concentration of 0.5 µM. Each bar represents an average of three biological replicates and the error bars show SD (*P < 0.05, ***P < 0.005)

Without artificial quinone electron acceptors, photosynthetic oxygen evolution, measured on Chl basis, was twice as fast in EL as in control cultures (Fig. 4a). When measured on per OD_730_ basis, approximating the relative rates per cell (the average cell density of the samples was 4.07 ± 0.059 × 10^6^ cells ml^−1^), the light-saturated rate of photosynthetic oxygen production turned out to be faster in the control than in the EL cells instead (Fig. 4b). However, very low oxygen evolution rates were measured from the EL cells when artificial PSII electron acceptor quinones of any kind were used. The highest rate was obtained with DMBQ whereas rates measured with the standard combination of DCBQ and FeCy, where the latter is included to keep DCBQ oxidized, yielded a very low rate (Fig. 4). DCBQ-dependent oxygen evolution continued both in control and EL cells upon addition of DBMIB, an inhibitor of oxidation of PQH_2_ at the cytochrome *b*_6_/*f* complex; in fact, a higher rate was measured from control cells in the presence of both DCBQ and DBMIB than with DCBQ alone. Photosynthetic oxygen evolution, measured without artificial PSII electron acceptors, was effectively inhibited in vivo by 0.5 μM DBMIB in both types of cells.

To test if the tested artificial quinones simply cannot penetrate to EL cells, we also measured oxygen evolution from isolated thylakoids. These measurements showed similar results as in vivo, as thylakoids isolated from EL cells produced less oxygen than control cells with both quinone electron acceptors (Fig. 4c), although the difference between control and EL thylakoids was less drastic than that between control and EL cells (Fig. 4).

### EL acclimation changes Chl a fluorescence kinetics

**Fig. 5:**
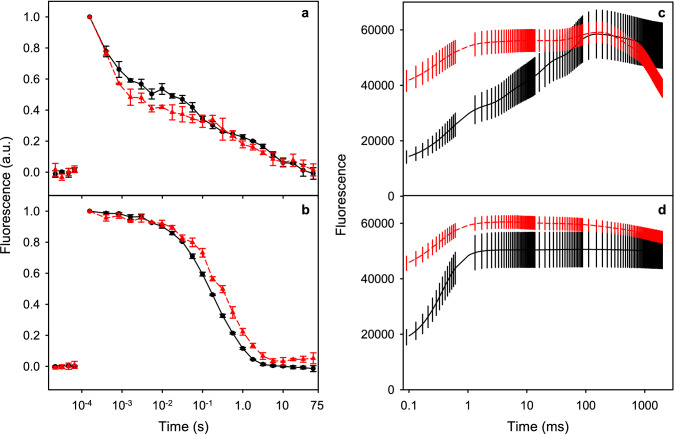
Decay of Chl *a* fluorescence yield after a single turnover flash (**a, b**) and fluorescence signal of Chl *a* fluorescence induction (**c, d**) measured in the absence (**a**, **c**) and presence (**b, d**) of DCMU from control (black, solid lines) and EL (red, dashed lines) *C. reinhardtii* cells in ambient air. Fluorescence measurements were conducted with 2 ml samples of cells containing either 5 µg Chl ml^−1^ (fluorescence decay, panels **a**, **b**) or 1 µg Chl ml^−1^ (fluorescence induction, panels **c**, **d**). The samples were dark-incubated for 15 min before measurement. The values in **a**, **b** are double normalized, first to the zero fluorescence level, measured before the flash, and then to the maximum fluorescence after the single turnover flash given at *t* = 1 ms. Fluorescence induction (**c**, **d**) was induced with blue, 455 nm actinic light with PPFD 400 µmol m^−2^ s^−1^. All curves are averaged from three independent biological replicates and the error bars show SD

A single turnover flash causes electron transfer to the Q_A_ quinone of PSII, and the fluorescence yield after the flash probes the oxidation of Q_A_^−^. The first, most rapid phase of the decrease in Chl *a* fluorescence yield after the single turnover flash was larger in EL samples than in the control samples (Fig. 5a). When the samples were supplemented with DCMU, an inhibitor that blocks electron transfer from Q_A_ to Q_B_, EL cells showed slower decrease of fluorescence than the control cells (Fig. 5b). The rate constant of recombination of the S_2_Q_A_^–^ state, obtained by fitting the curves measured in the presence of DCMU to a first-order equation, was 0.24 s^−1^ for the control and only 0.08 s^−1^ for the EL cells.

Chl *a* fluorescence induction was very different in EL cells than in the control cells (Fig. 5c). When fluorescence induction was measured in the absence of DCMU, EL cells had a much higher *F*_0_ level but a similar *F*_M_ level as control cells, and consequently the *F*_V_/*F*_M_ value of the EL cells (0.30 ± 0.04) was much lower than that of the control cells (0.76 ± 0.00) (Fig. 5c). In the presence of DCMU, lower *F*_V_/*F*_M_ values were obtained from both types of cells than in the absence of DCMU, but the difference between EL and control cells remained similar as in the absence of DCMU. A decrease in fluorescence yield after the maximum was observed in the EL cells both in the absence and presence of DCMU (Fig. 5c, d).

Examination of the OJIP kinetics shows that in EL cells, the O-J-transition comprised most of the initial fluorescence rise and no J-I-transition could be resolved (Fig. S2), whereas the control cells expressed standard behavior of fluorescence induction. In the absence of DCMU, both types of cells showed maximal fluorescence at the same time point of 161 ms. In the presence of DCMU, fluorescence yield was higher in EL than in control cells (Fig. 5d).

### Acclimation induces minor changes in thermoluminescence

**Fig. 6:**
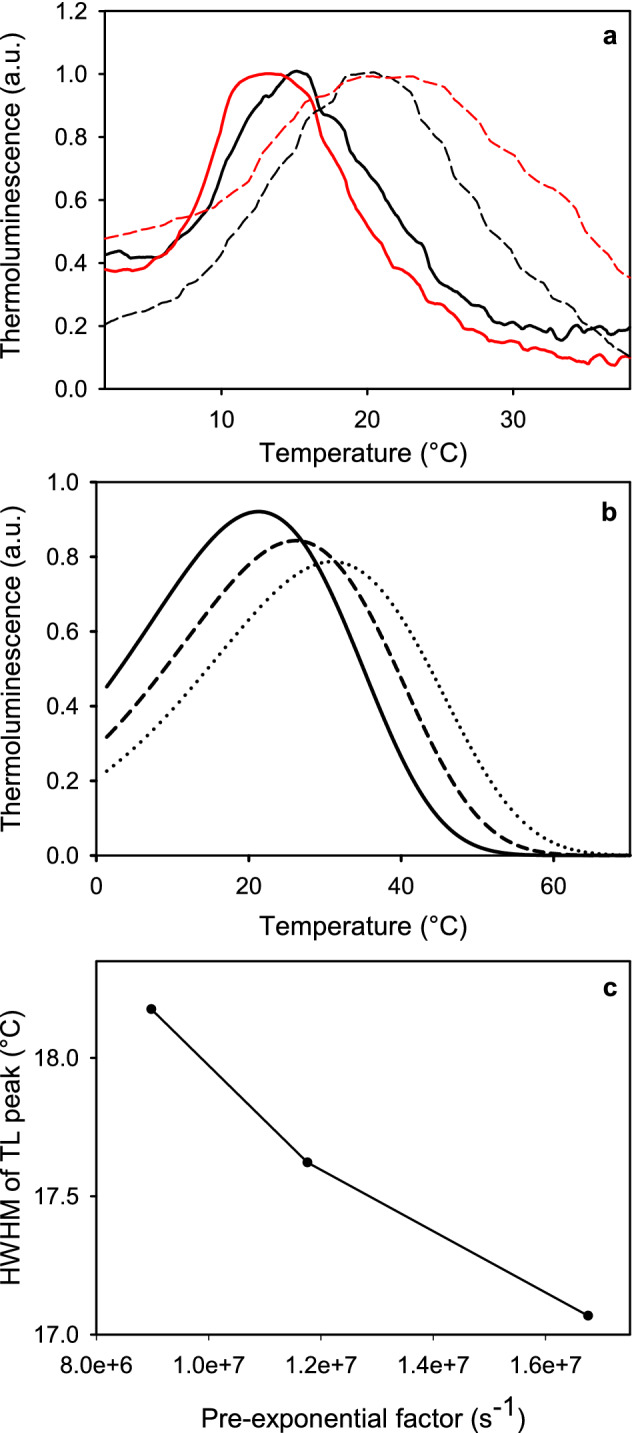
**a** Thermoluminescence Q band (solid line) and B band (dashed line) measured from control (black) and EL (red) cultures of *C. reinhardtii*. 200 µl samples, containing 3.1 µg of Chl, were cooled down to either − 20 °C (for Q band measurements) or − 10 °C (B band measurements) and then charged with a single turnover flash. The temperature was then gradually increased up to 60 °C at a heating rate of 0.66 °C s^−1^. The Q bands were recorded in the presence of 20 µM DCMU. All bands are averaged from three biological replicates. **b** Simulated thermoluminescence curves assuming a first-order reaction with *E*_a_ = 501 eV, heating rate 0.66 °C s^−1^ and pre-exponential factor of Arrhenius’ equation of 1.7 × 10^7^ s^−1^ (solid line), 1.2 × 10^7^ s^−1^ (dashed line) or 9 × 10^6^ s^−1^ (dotted line) and **c** the half width at half maximum of the thermoluminescence band as a function of the pre-exponential factor

The slow recombination of the S_2_Q_A_^−^ state prompted us to use thermoluminescence to measure eventual differences in the redox potentials of the PSII electron acceptors. The thermoluminescence B band is associated with the S_2,3_Q_B_^−^ → S_1,2_Q_B_ recombination and the Q band with the S_2,3_Q_A_^−^ → S_1,2_Q_A_ recombination. The Q band peaked at 13.6 °C in EL cells and 15.5 °C in control cells (Fig. 6), and the B band of the EL cells peaked at 21.6 °C, whereas the B band of control peaked at 20.0 °C. Furthermore, the B band of the EL cells was wider than that of the control cells. The quality of the thermoluminescence data did not allow fitting, but a simulation of the behavior of a first-order thermoluminescence band showed that a lower pre-exponential factor of Arrhenius's equation might explain why the B band of the EL cells was wider and shifted to a higher temperature, compared to control cells (Fig. 6b, c).

### EL acclimated cells produce less singlet oxygen than control cells

**Fig. 7:**
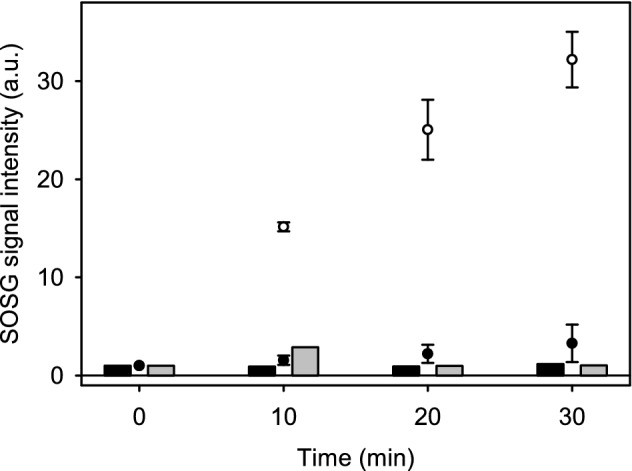
^1^O_2_ production, measured as increase of fluorescence emitted by endoperoxidized SOSG, of control (open circles) and EL cells (black circles) in comparison to light controls illuminated without SOSG (grey bars) and dark controls (black bars) incubated in the dark with SOSG. 350 µl samples containing 50 µg ml^−1^ Chl were supplemented with 50 µM SOSG and then treated with red light (> 650 nm) with PPFD 2000 µmol m^−2^ s^−1^. The fluorescence emitted by SOSG that had reacted with ^1^O_2_ was excited with 500 nm light and recorded spectrophotometrically at 535–540 nm. Each data point represents an average of three biological replicates and the error bars show SD

Reactive oxygen species are produced in chloroplasts in the light, and especially in strong light, and ^1^O_2_ production by PSII depends on recombination reactions that lead to the triplet state of P_680_ (Krieger-Liszkay et al. 2008). The slow S_2_Q_A_^−^ recombination in the EL cells (Fig. 5b) might therefore predict a low ^1^O_2_ yield. We used SOSG to measure ^1^O_2_ production from control and EL cells. For the measurement, a cell suspension was supplied with SOSG and illuminated with high-intensity red light (PPFD 2000 μmol m^−2^ s^−1^, > 650 nm) that does not induce ^1^O_2_ production by SOSG itself (Hakala-Yatkin and Tyystjärvi 2011). Both control and EL cells produced ^1^O_2_ at essentially constant rates throughout the 30-min illumination period. However, the rate of ^1^O_2_ production by an EL cell suspension was only 10.2% of that of control cells, when suspensions containing the same amount of Chl were compared (Fig. 7).

### EL tolerance is not accompanied by a slow damaging reaction of photoinhibition

Effect of light on PSII activity was measured both in the presence and absence of lincomycin that blocks the repair of PSII. Cells were illuminated at PPFD 950 µmol m^−2^ s^−1^ and PSII oxygen evolution was measured from aliquots of the illuminated suspension with Chl concentrations of 8.93 ± 0.56 µg ml^−1^ and 4.87 ± 0.78 µg ml^−1^ for control and EL samples, respectively. DMBQ was used as electron acceptor. Again, DMBQ-dependent oxygen evolution, measured before the illumination treatment, was much slower in EL than in control cells.

**Fig. 8:**
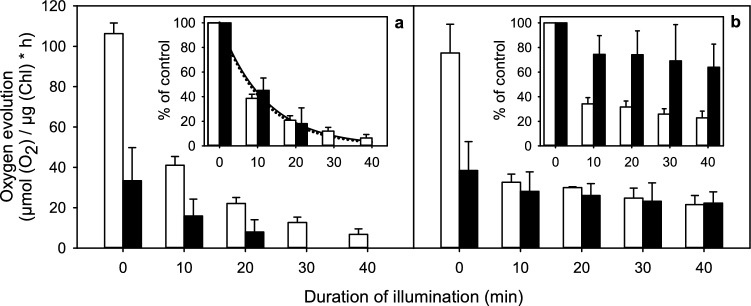
Photoinhibition of PSII in vivo measured as decrease in light-saturated oxygen evolution in control (white bars) and EL (black bars) cultures of *C. reinhardtii*, illuminated in the presence (**a**) and in the absence (**b**) of lincomycin. The insets show data from **a** and **b** when normalized to the control value at *t* = 0. Live cells, grown in either pre-culture or EL conditions, as indicated, were collected and used at OD_730_ of 0.5 (cell density 4 × 10^6^ cells ml^−1^, 8.9 µg ml^−1^ and 4.9 µg ml^−1^ Chl in control and EL samples, respectively). A 10 ml sample was treated with photoinhibitory white light (PPFD 950 µmol m^−2^ s^−1^) and a 1 ml aliquot was used to quantify the number of active PSII units in the illuminated sample by measuring the rate of light-saturated oxygen evolution (H_2_O to DMBQ). 1 ml aliquots were supplemented with DMBQ and FeCy in final concentration of 0.5 mM prior to measuring the oxygen evolution rate. The lines in inset of **a** represent the best fit to the reaction equation describing the kinetics of damaging reaction of photoinhibition. Each bar represents an average of three biological replicates and the error bars show SD

Illumination of cells in the presence of lincomycin led to a clear first-order decay of PSII oxygen evolution activity in both types of cells (Fig. 8a). The rate of loss of PSII activity was similar in both EL and control cells, with *k*_PI_ values of 1.47 ± 0.12 × 10^–3^ s^−1^ and 1.38 ± 0.09 × 10^–3^ s^−1^ in control and EL samples, respectively (Fig. 8a, inset). Thus, the damaging reaction of photoinhibition had the same rate in EL and control cells.

When the loss of active PSII units was measured under the same PPFD in the absence of lincomycin, EL cells were, as expected, hardly affected (Fig. 8b, inset). Control cells, in turn, rapidly lost PSII activity, and already after 20 min of illumination, both cell types had roughly the same rate of oxygen production (Fig. 8b). Finally, the damaging reaction and repair of PSII equilibrated in both control and EL samples to a similar oxygen production rate, 21.57 ± 4.5 and 22.29 ± 5.6 µmol (O_2_) µg (Chl)^−1^ h^−1^ for control and EL cells, respectively. The result may suggest that the low ^1^O_2_ production of the EL cells exerts its advantageous effect on translation of chloroplast proteins (Nishiyama Y et al. 2001) only during a long cultivation in EL but not yet during a short-time photoinhibition experiment.

## Discussion

### How does *C. reinhardtii* turn tolerant to an extreme light intensity

Effects of exposure of photosynthetic organisms to high light for a few hours has been studied extensively (for review, see Tyystjärvi 2013), and also acclimation to strong but not extreme light is a thoroughly studied topic in both plants and green algae (Bonente et al. 2012; Kouřil et al. 2013; Dietz 2015; Belgio et al. 2018; Virtanen et al. 2019). However, less is known about how organisms cope with prolonged exposure to light intensities highly exceeding full sunlight. In fact, it was only recently shown that the optimum PPFD for biomass production by *C. reinhardtii* is closer to 800 μmol m^−2^ s^−1^ (Virtanen et al. 2019) than the moderate PPFD values of 80–200 μmol m^−2^ s^−1^ usually applied in cultivation of the alga. Here we examined how wild-type *C. reinhardtii* reacts when suddenly transferred to PPFD 3000 μmol m^−2^ s^−1^ after precultivation at PPFD 100 μmol m^−2^ s^−1^.

The first finding was that although *C. reinhardtii* stops growing when suddenly exposed to extreme light intensity, growth resumes after a few days in the majority of culture bottles (Fig. 1a). The phenomenon was found to be so common, even when using cultures originating from single cells as inocula (Table 1), that mutation can be excluded as a cause. The finding that the rapidly obtained high-light tolerance is tuned down in a week, and in some cases even lost, supports this conclusion (Table 1, Fig. 1b). However, this finding does not exclude the possibility that acclimation to high light by mutations also occurs, as reported in earlier publications (Förster et al. 2005; Schierenbeck et al. 2015).

The finding that isolated subpopulations acclimate more rapidly and, in terms of growth, remain 24 h ahead of the cultivations started from individual cells, suggests that acclimation to extreme light has a genetic/epigenetic component. Actual genetic variation within a *C. reinhardtii* culture is unlikely, as the maintenance cultivation does not induce sexual reproduction, making epigenetic modification a more appealing explanation. Epigenetic regulation is also in line with the slowly reversible nature of the observed, EL acclimated state. Epigenetic differences might be induced by subtle differences in the interplay between the environment and developmental phase between individual cells during maintenance. It has already been shown that the amount of epigenetic variation in a *C. reinhardtii* population can contribute to its capability to acclimate to different environmental stress factors (Kronholm et al. 2017; Duarte-Aké et al. 2018). In addition, the chloroplast genome of *C. reinhardtii* has been reported to be especially prone to modification via methylation by DNA methyltransferase DMT 1 (Nishiyama R et al. 2002). It is also possible that the methylation states of key genes change occur during the acclimation period, as the time window of epigenetic regulation in *C. reinhardtii* (Umen and Goodenough 2001) matches with the time that it takes for cultures to start growing in EL.

### Physiological features of EL acclimated cells

The differences in the properties of *C. reinhardtii* cells before and after EL acclimation may obviously reflect stress, acclimation, or both. The decrease in the Chl content of the cells might indicate reduction in the antenna size that, in turn, would reduce the so-called excess excitation energy absorbed by the photosynthetic machinery (Öquist et al. 1993). The change in the Chl *a*/*b* ratio would indicate an alteration in the functional size of the antenna (Kirst et al. 2012). However, this ratio remains constant throughout the acclimation (Fig. 2b), suggesting that the EL acclimated cells retain the functional size of their antennae. In agreement with the stable Chl *a*/*b* ratio, the finding that the amounts of photosystems decrease during EL acclimation approximately as much as the amount of chlorophyll (Figs. 2, 3) suggest that the overall number of photosystems per cell decreases during EL acclimation but the amount of Chl associated with each photosystem remains stable.

In addition to the overall decrease of the photosystems, the 77 K fluorescence (Fig. 1c) and Western blot data (Fig. 3) suggest that PSII units decrease more than PSI units. The most straightforward interpretation for the low amount of PSII in the EL cells is that the EL treatment causes so rapid photoinhibition that the repair of PSII fails to maintain full activity, and eventually some PSII units become completely degraded. This interpretation is in agreement with the finding that the rate of the damaging reaction of photoinhibition of PSII is the same in EL and control cells (Fig. 8a, inset). Furthermore, the fluorescence induction data show that most PSII centers of the EL cells are in an inactive, photoinhibited state, as the F_V_/F_M_ ratio is very low and the OJIP curves closely resemble curves obtained in the presence of DCMU (Fig. 5c, d). In addition, the finding that the amount of PQ per cell shows a slight increase during EL acclimation (Fig. 1d) suggests that the number of PSII units decreases without a simultaneous change in the amount of plastoquinone in the thylakoid membranes.

Intriguingly, these data are contrary to what happens in *C. reinhardtii* during a long-time acclimation to high but not extreme light intensity (Bonente et al. 2012; Virtanen et al. 2019), where the ratio of PSII to PSI increases, indicating a more drastic decrease in PSI than in PSII units. However, here we observe also decrease in PSI content (Fig. 3). In addition, LHCSR3 accumulates in high light (Tibiletti et al. 2016), which is most probably the case also in EL conditions, where it protects both photosystems by inducing NPQ (Girolomoni et al. 2019). The combination of photoinhibition and NPQ probably cause PSII fluorescence to decrease more than PSI fluorescence during EL treatment and acclimation.

Drastic decrease in the rate of production of ^1^O_2_ in extreme light (Fig. 7) is most obviously a high-light acclimation response. ^1^O_2_ is mainly generated by PSII (Krieger-Lizkay 2005; Telfer 2014) and besides functioning as a general agent of harmful oxidation, ^1^O_2_ is known to specifically oxidize cyanobacterial translation elongation factors (Kojima et al. 2007), which suggests that ^1^O_2_ slows down PSII repair also in chloroplasts by interfering with chloroplast protein synthesis (Nishiyama Y et al. 2001; Hakala-Yatkin et al. 2011). ^1^O_2_ has also been suggested to directly damage PSII in photoinhibition (Vass 2011). However, the low ^1^O_2_ levels in EL cells did not slow down the damaging reaction of photoinhibition in EL *C. reinhardtii* (Fig. 8), suggesting that ^1^O_2_ is not a crucial factor in determining the rate of photoinhibition.

Slow ^1^O_2_ production is a common feature in both EL acclimated *C. reinhardtii* and *Chlorella ohadii*, a green alga famous for being resistant both to strong light and photoinhibition of PSII (Treves et al. 2016). However, the mechanisms of high-light tolerance in these two species of algae are most probably different. In *C. ohadii*, the PSII antenna is small and the charge recombination reactions are less likely to produce triplet states than in PSII found in other autotrophs (Treves et al. 2016). The thermoluminescence data from EL cells of *C. reinhardtii*, in contrast, indicate neither a decrease in the redox potential gap between Q_A_/Q_A_^−^ and Q_B_/Q_B_^−^ pairs nor a positive shift in the potential of the Q_A_/Q_A_^−^ pair (Fig. 6; see Rappaport et al. 2002 for the general interpretations), suggesting that the probability of triplet formation by PSII recombination reactions is not altered by EL acclimation. However, the charge recombination reactions themselves are affected, as the S_2_Q_A_^−^ → S_1_Q_A_ recombination appears to be slower in EL than in control cells (Fig. 5b), which may at least partly explain the low ^1^O_2_ production rate. The slower rate of recombination may, at least partially, be caused by a change in the pre-exponential factor of the Arrhenius equation of the rate constant of recombination (Fig. 6). A small pre-exponential factor would simply slow down the rate of S_2_Q_A_^−^ → S_1_Q_A_ recombination in EL cells, in comparison to control cells. PSII units of both a wild-type organism (Treves et al. 2016) and mutants (Fufezan et al. 2007) have been shown to be functional in spite of structural differences that cause variations in the redox potentials of PSII electron acceptors. Therefore, acclimation-dependent changes affecting the pre-exponential factor would not be surprising. Another obvious feature, and possibly more prominent one, to explain the low levels of ^1^O_2_ in EL cells is their very high carotenoid-to-Chl ratio (Fig. 2c). Carotenoids are important scavengers of ^1^O_2_ (Ramel et al. 2012), and may quench ^1^O_2_ before it can be detected by a reaction with SOSG to a degree. The sum of these two factors could be the cause for the observed results from SOSG-dependent detection of ^1^O_2_.

Interestingly, acclimation to EL is associated with a decreased ability to reduce artificial quinone electron acceptors (Fig. 4), suggesting that the side chain of PQ is important for maintaining a sufficient rate of electron transfer to PQ in EL cells. The rates of electron transfer to artificial quinone electron acceptors are slower than photosynthesis in EL cells, indicating that the tested artificial quinones, in addition to acting as poor electron acceptors of PSII in the EL acclimated cells, also inhibit electron transfer to the natural PQ. The behavior of the artificial quinones can be flexible, as e.g. DBMIB is known to be able to bypass its own blockage and act also as an electron acceptor in vitro (Schansker et al. 2005). In vivo, however, DBMIB primarily caused cessation of electron transfer from PSII and only oxygen consumption was observed in its presence. Furthermore, 2,5-dimethylbenzoquinone, a sister compound of DMBQ used here (2,6-dimethylbenzoquinone), interacts less strongly with the Q_B_ binding site of PSII than DCBQ (Graan and Ort 1986), which may explain why a higher electron transfer rate was obtained with DMBQ than with DCBQ.

The oxygen evolution measurements from isolated thylakoids confirmed that the slow rates of electron transfer to the quinone acceptors was not caused by slow diffusion of the quinones to EL acclimated cells. Furthermore, as the chlorophyll concentrations of the samples in the in vitro experiments were the same, the number of PSII units in control and EL acclimated samples was the same. Thus, the rate of oxygen evolution per PSII unit was also much slower in EL cells than in the control cells in vitro. Together all these data strongly suggest that PSII has changed in the acclimation process. We hypothesize that EL acclimation causes subtle structural or conformational changes in PSII, causing the observed changes in reduction of artificial quinone electron acceptors. It is tempting to also hypothesize that the differences observed in the function of the acceptor side might be related to the slow rate of charge recombination in the PSII of the EL cells.

Comparison of EL and control cells during high-light illumination in the absence of lincomycin shows that the level at which PSII activity equilibrates in high light is not higher in EL than in control cells (Fig. 8b). This equilibrium is reached as a result of concurrent damaging and recovery reactions (Samuelsson et al. 1985; Greer and Laing 1988; for a review, see Campbell and Tyystjärvi 2012), and the similarity of the equilibrium levels in EL and control cells indicates that the recovery reactions run at the same rate in both cell types, when measured on chlorophyll basis. However, during EL exposure, proportionally low PSII activity may be high enough to support cellular functions, as light intensity is not limiting.

The Q_A_^−^Q_B_ → Q_A_Q_B_^−^ electron transfer reaction seems to be faster in EL than in control cells (Fig. 5a), and the light-saturated rate of photosynthesis, with PQ as the electron acceptor of PSII, is faster in EL than in control cells if measured on per Chl basis (Fig. 4). However, on a per cell basis, the rate of photosynthesis of EL cells appears to be slower than in control cells, as the amount of Chl per cell decreases to one half during the EL acclimation (Fig. 2a).

The fluorescence induction curves (Fig. 5c) of EL cells reveal a rapid decrease in fluorescence yield right after the peak fluorescence value. A similar although milder response is seen in the presence of DCMU (Fig. 5d). Induction of LHCSR3-dependent non-photochemical quenching may explain why fluorescence yield decreases in the absence of DCMU, as this type of NPQ takes more than the applied 15 min dark-incubation to relax (Allorent et al. 2013). The decrease in the presence of DCMU, in turn, might reflect light-induced enhancement of fluorescence quenching by the inactive, severely photoinhibited PSII centers. The overall reduction of PSII electron acceptors during OJIP measurements occurs at similar pace in both types of cells, indicated by the similar time it took the fluorescence to reach the F_M_ level (Fig. 5c). These data add further evidence for the suggestion that PSII is a target of the EL acclimation process.

## Conclusions

The results of the present study show that a culture of wild-type *C. reinhardtii* cells can rapidly acclimate to extreme PPFD as high as 1.5 times direct sunlight, or 10–20 times the usual cultivation PPFD. The acclimation mechanism shows signs suggesting involvement of epigenetic variation present in the algal population. The EL acclimated phenotype has less both photosystems per cell and a higher carotenoid-to-chlorophyll ratio than the control cells. Furthermore, PSII charge recombination reactions in EL acclimated cells are slow, possibly due to conformational changes that affect the pre-exponential Arrhenius factor of the rate constant of charge recombination, rather than changes in redox potentials of the electron carriers. Slow charge recombination and high carotenoid-to-Chl ratio probably explain why the EL cells also show a low ^1^O_2_ production rate. Low rate of ^1^O_2_ production in high light is expected to keep the recovery of photoinhibited PSII functional during growth in EL. On the other hand, the rate of the damaging reaction of photoinhibition of PSII is similar in EL acclimated and control cells.

## Electronic supplementary material

Below is the link to the electronic supplementary material.

Electronic supplementary material 1 (PDF 171 kb)

## Data Availability

A dataset is available at https://data.mendeley.com/datasets/fwnzkph5w2/draft?a=bd44a7ec-58544a-4e7b-9043-f773adf5065b.
