# Supplementary material for: Acclimation of Chlamydomonas reinhardtii to extremely strong light
Source: Photosynth Res. 2020 Dec 6;147(1):91–106. doi: 10.1007/s11120-020-00802-2 (PMC7728646; doi:10.1007/s11120-020-00802-2)
Supplement: Supplementary file 1 — Electronic supplementary material 1 (PDF 171 kb) [file 11120_2020_802_MOESM1_ESM.pdf]

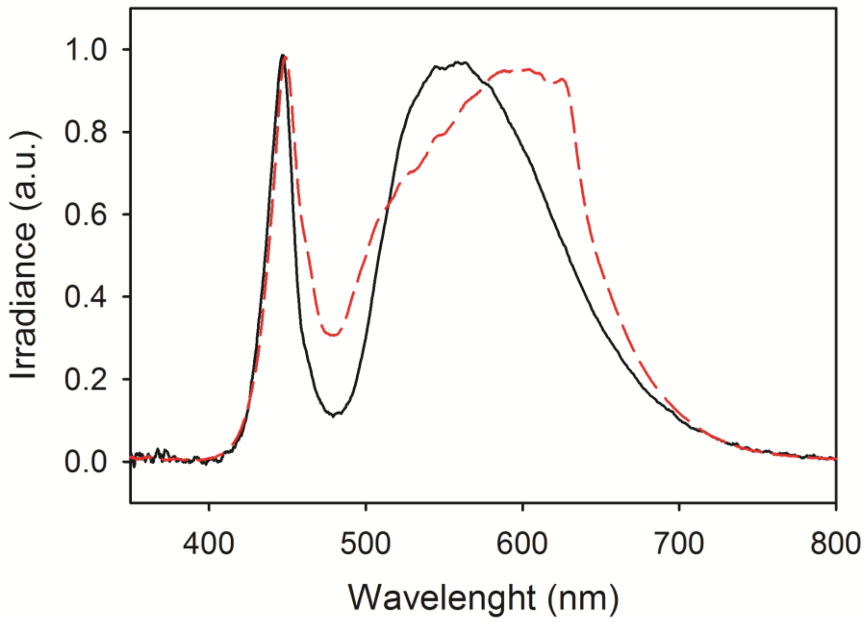

Fig. S1 Spectra of the lights used in preculturing (black, solid line) and extreme light (red, dashed line) conditions. The spectra were measured with a calibrated spectrometer (Ocean Insight, STS-VIS) and normalized to the maximum at 448 nm.

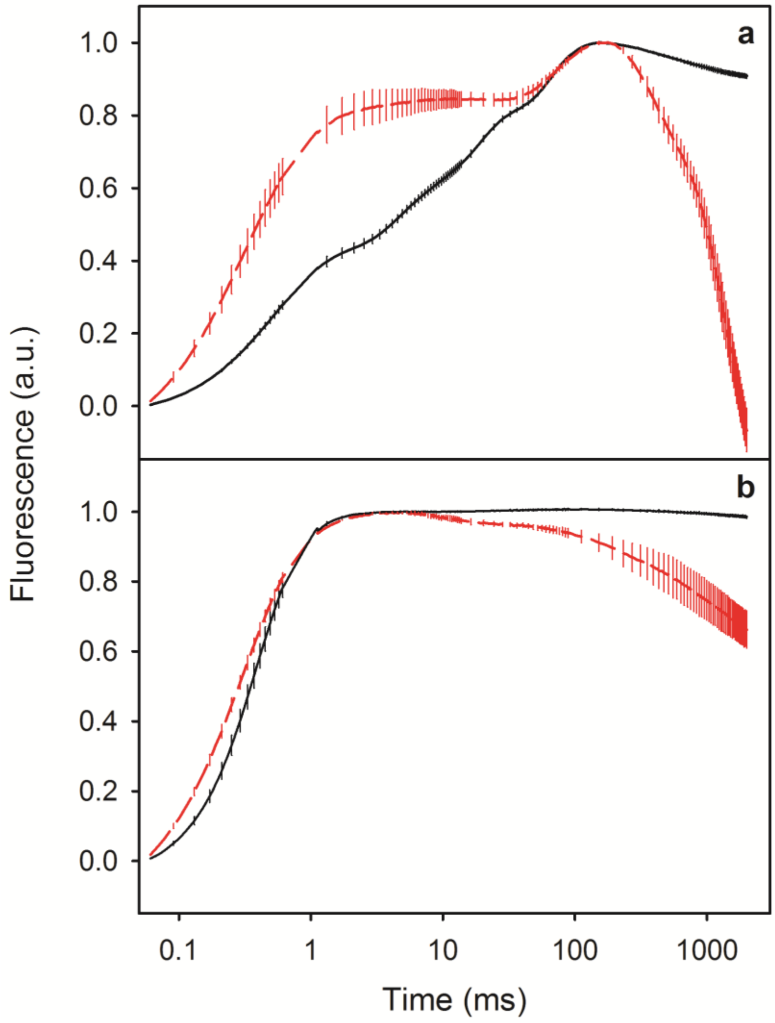

Fig. S2 Chl *a* fluorescence induction in the absence (**a**) and presence (**b**) of DCMU in control (black, solid line) and EL cells (red, dashed line), double normalized, first to the zero fluorescence level and then to the maximum fluorescence values at either 161 ms (**a**) or 4.4 ms (**b**) The curves are averaged from three independent biological replications and the error bars show SD.
